# Lymphatic Endothelial Markers and Tumor Lymphangiogenesis Assessment in Human Breast Cancer

**DOI:** 10.3390/diagnostics12010004

**Published:** 2021-12-21

**Authors:** Jia-Mei Chen, Bo Luo, Ru Ma, Xi-Xi Luo, Yong-Shun Chen, Yan Li

**Affiliations:** 1 Center of Oncology, Renmin Hospital of Wuhan University, Wuhan 430060, China; chenjiamei@whu.edu.cn (J.-M.C.); xixiluo@whu.edu.cn (X.-X.L.); 2 Department of Pathology, The Central Hospital of Wuhan, Tongji Medical College, Huazhong University of Science and Technology, Wuhan 430014, China; luobo198705051@126.com; 3 Department of Peritoneal Cancer Surgery, Beijing Shijitan Hospital of Capital Medical University, Beijing 100038, China; maru1005@mail.ccmu.edu.cn; 4 Department of Pathology, Beijing Shijitan Hospital, Capital Medical University, Beijing 100038, China

**Keywords:** breast cancer, lymphatic endothelial cell, markers, lymphangiogenesis, lymphatic vessel density, lymphatic vessel invasion

## Abstract

Metastasis via lymphatic vessels or blood vessels is the leading cause of death for breast cancer, and lymphangiogenesis and angiogenesis are critical prerequisites for the tumor invasion–metastasis cascade. The research progress for tumor lymphangiogenesis has tended to lag behind that for angiogenesis due to the lack of specific markers. With the discovery of lymphatic endothelial cell (LEC) markers, growing evidence demonstrates that the LEC plays an active role in lymphatic formation and remodeling, tumor cell growth, invasion and intravasation, tumor–microenvironment remodeling, and antitumor immunity. However, some studies have drawn controversial conclusions due to the variation in the LEC markers and lymphangiogenesis assessments used. In this study, we review recent findings on tumor lymphangiogenesis, the most commonly used LEC markers, and parameters for lymphangiogenesis assessments, such as the lymphatic vessel density and lymphatic vessel invasion in human breast cancer. An in-depth understanding of tumor lymphangiogenesis and LEC markers can help to illustrate the mechanisms and distinct roles of lymphangiogenesis in breast cancer progression, which will help in exploring novel potential predictive biomarkers and therapeutic targets for breast cancer.

## 1. Introduction

The latest data show that breast cancer has become the most common malignant carcinoma, with 2.26 million new cases in 2020 worldwide [1]. Metastasis via lymphatic vessels or blood vessels is the leading cause of death for breast cancer, and tumor lymphangiogenesis and angiogenesis are critical prerequisites for the tumor invasion–metastasis cascade [2,3,4]. Whether breast cancer cells gain access to the systematic circulation via blood vessels or via lymphatic vessels at the primary tumor site remains an open question [3,5,6,7]. It is well known that angiogenesis is a critical process for cancer growth, and anti-angiogenic therapy has been incorporated into treatment guidelines for some solid tumors [8]. As early as 1996, an international consensus on the quantification of angiogenesis was established and was updated five years later [9]. Conversely, research progress for lymphangiogenesis has lagged behind that for angiogenesis due to a lack of specific markers. There were few studies about tumor lymphangiogenesis, until the discovery of lymphatic markers that can distinguish lymphatic vessels from blood vessels or other structures, which is now significantly driving the progression of tumor lymphangiogenesis research [10,11,12,13,14].

Studies reveal that tumor lymphangiogenesis is involved in cancer progression [6,7,11,12,13]. Mouse models have shown that the blood vessels in lymph nodes can serve as a short-cut route for tumor cells entering the systemic circulation, which also challenges the assumption that lymph node involvement in breast cancer is an epi-phenomenon [6,7]. Beyond providing a circulation conduit for tumor cells, growing evidence demonstrates that tumor lymphatic endothelial cells (LECs) are actively involved in lymphatic formation and remodeling, tumor cell growth, tumor- and immune-cell-directed invasion, antitumor immunity, the tumor microenvironment, and premetastatic niches remodeling [12,13,14,15]. A high lymphatic vessel density (LVD) was found to correlate with lymph node metastasis or poor survival in breast cancer [16]. Ndiaye et al. [17] indicated that the lymphatic vessel is a double-edged sword in tumor metastasis. In addition, Niemiec et al. [18] found that a high LVD identified a high risk of progression in pN0/chemotherapy-naive patients but low risk in pN+/chemotherapy-treated patients. Ginter et al. [19], however, found that lymphatic vessels were occasionally present in tumor cell nests or tumor-associated stroma and did not participate in metastasis. These discrepant findings may be partly attributable to the difference in the lymphatic markers and stains used, inconsistent counting methods for LVD, and the distinct subpopulations of patients across studies.

In this review, we summarized recent findings on tumor lymphangiogenesis, the most commonly used LEC markers, and parameters for lymphangiogenesis assessment, such as the LVD and lymphatic vessel invasion (LVI) in human breast cancer. A profound understanding of tumor lymphangiogenesis and LEC markers could help to delineate the mechanisms and distinct roles of lymphangiogenesis in breast cancer progression, facilitating the exploration of novel potential predictive biomarkers and therapeutic targets for breast cancer.

## 2. Tumor Lymphangiogenesis

Tumor lymphangiogenesis is the formation of new lymphatic vessels [4,20], that generally constitute lymphatic capillaries or initial collecting lymphatics, a crucial initiating step in tumor spread.

### 2.1. Lymphatic Capillaries and Collecting Lymphatics

**Figure 1:**
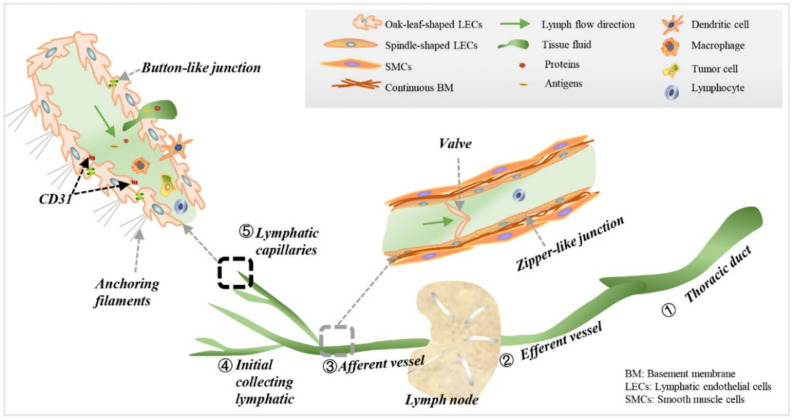
Tree-shaped lymphatics are composed of lymphatic capillaries, initial collecting lymphatics, afferent and efferent lymphatics, and the thoracic duct. Lymphatic capillaries are expanded blind-ended irregular lumens lined with a single layer of oak-leaf-shaped LECs without pericytes or SMCs, and anchored to the surrounding tissue via anchoring filaments. There is no BM around the capillaries. Overlapping flaps between adjacent oak-leaf-shaped LECs are anchored on the sides by discontinuous button-like junctions. CD31 is located at the tip of flaps, where there is a lack of buttons. By contrast, collecting lymphatics are lined with spindle-shaped LECs anchored by zipper-like junctions and covered with continuous BM and SMCs. Gaps at overlapping flaps in lymphatic capillaries are lymph inlets, while valves in collecting lymphatics are backflow-prevention structures.

Lymphatics are tree-shaped hierarchical networks composed of lymphatic capillaries, initial collecting lymphatics, afferent and efferent collecting lymphatics, and the thoracic duct [21] (Figure 1). Generally, lymphatic capillaries are expanded blind-ended irregular lumens lined with a single layer of oak-leaf-shaped LECs and anchored to the surrounding tissue via anchoring filaments [21,22]. In lymphatic capillaries, overlapping flaps (i.e., primary lymphatic valves) between adjacent LECs are anchored on the sides by discontinuous button-like junctions [23]. By contrast, the collecting lymphatics are lined with elongated spindle-shaped LECs with continuous zipper-like junctions and covered with continuous basement membrane (BM) and a smooth muscle cells layer, which helps to pump the lymph [23]. Both button- and zipper-like junctions are composed of vascular endothelial cell cadherin (VECD) and tight junction proteins [23]. In addition, the platelet/endothelial cell-adhesion molecule-1, namely CD31, is partially colocalized with VE-cadherin at zipper-like junctions, and at the tip of overlapping flaps where there is a lack of buttons [21,23]. Moreover, there are no pericytes or smooth muscle cells covering the LEC layer of lymphatic capillaries. In addition, the BM around the capillaries is always absent or discontinuous. The anatomical features mentioned above facilitate the entry of tissue fluid, proteins, antigens, tumor and immune cells into the capillaries through gaps between buttons located at the overlapping flaps [24,25,26].

Crosstalk between lymphatics and immune cells plays an important role in the uptake of cells into lymphatics [25]. Tumor cells can actively enter the lymphatics via chemokine gradients generated by LECs [26,27]. Multiple mechanisms of directed cell migration have been thoroughly reviewed [28]. Then, the afferent collecting lymphatics drain lymph into lymph nodes, where the adaptive immune response and immune tolerance are initiated. Overlapping flaps in lymphatic capillaries facilitate lymph entry, while valves (i.e., secondary lymphatic valves) in collecting vessels maintain the lymph flow in one direction and prevent backflow. In addition, initial collecting lymphatics or precollectors, located between lymphatic capillaries and collecting lymphatics, are composed of a single layer of endothelia, secondary valves, and continuous CD31-VECD junctions [21]. Through these mechanisms, the lymphatics play an important role in maintaining the homeostasis of body fluids and immunity.

### 2.2. Tumor Lymphangiogenesis and Lymphatic Endothelial Cellular Origins

**Figure 2:**
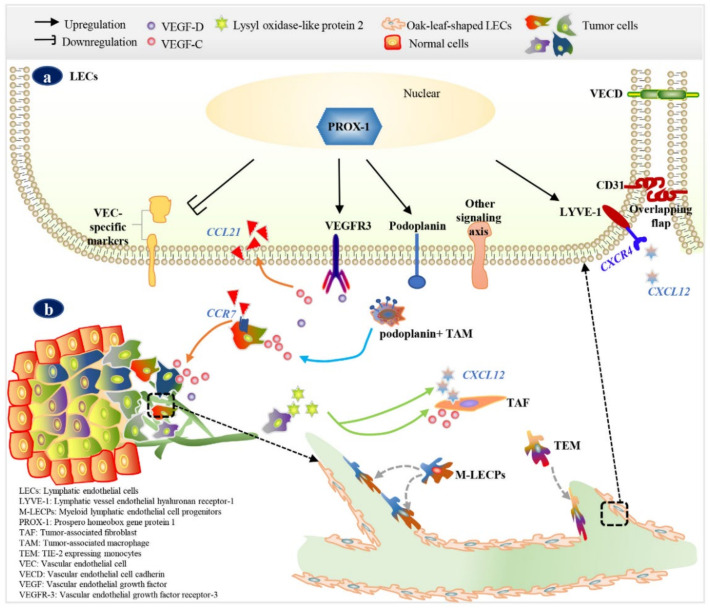
Lymphangiogenesis and lymphatic endothelial cellular origins in breast cancer. (**a**) LEC precursors are polarized by the specific expression of PROX-1 in nuclei; then, the expression of VEGFR-3, LYVE-1, and podoplanin on the cell membrane is up-regulated and the expression of VEC-specific genes is down-regulated. (**b**) Among multiple signaling axes, VEGF-C/VEGF-D/VEGFR-3 is the central axis promoting LEC proliferation, migration, and survival. Podoplanin-expressing TAMs [42] and the CCL21/CCR7 chemokine axis [44] might mediate VEGF-C secretion by tumor cells and stimulate lymphangiogenesis. Additionally, tumor cells expressing lysyl oxidase-like protein 2 might enhance lymphangiogenesis via stimulating VEGF-C and CXCL12 secretion by TAFs [46] and activating the CXCR4/CXCL12-LYVE-1 axis [45]. The majority of tumor LECs sprout from pre-existing lymphatic vessels; a few LECs were found to originate from bone marrow-derived cell progenitors, such as M-LECPs [36] and TEMs [49].

The transcription factor prospero homeobox gene protein 1 (PROX-1), a homologue of the *Drosophila melanogaster* PROX-1, is known to be a master control gene for lymphatic differentiation and subsequent vasculature formation [29,30]. According to the venous origin theory, LECs could originate from a subpopulation of vascular endothelial cells (VECs) in the cardinal vein (CV) or the LEC progenitors in the intersegmental vessels during embryogenesis [31,32,33]. PROX-1-expressing endothelial cells (ECs) acquire a lymphatic identity resulted in down-regulation of VEC-specific markers and up-regulation of LEC-specific markers such as vascular endothelial growth factor receptor-3 (VEGFR-3) and lymphatic vessel endothelial hyaluronan receptor-1 (LYVE-1) [29,30] (Figure 2a). Subsequently, the differentiated LECs sprout out of the CV and migrate towards the signal site finely modulated by stimulators and suppressors. In PROX-1-null mice, the ECs in the CV did not express LEC markers or secrete secondary cytokines [34]. Regarding tumor lymphangiogenesis, earlier studies stated that tumor-associated LECs originate from the existing lymphatics. With the deepening of research, more and more studies demonstrated that some tumor-associated LECs showed non-venous origins, such as mesenchymal origin [32,35,36,37].

Tumor lymphangiogenesis is a complex process mediated by multi-functional cytokines secreted by tumor cells and cells in the tumor microenvironment [38,39] (Figure 2b). Among which, the vascular endothelial growth factor (VEGF)-C is the first isolated and the most well studied pro-lymphangiogenic factor [40]. VEGF-C/VEGF-D/VEGFR-3 is the central and classical signaling axis currently known to promote LECs proliferation, migration, and survival [41]. It was reported that podoplanin-expressing tumor-associated macrophages could stimulate lymphangiogenesis via activating VEGF-C/VEGFR-3 or integrin pathways [42,43]. In addition, the CCL21/CCR7 chemokine axis was shown to mediate VEGF-C secretion of breast tumor cells [44]. Some other signaling pathways are probably involved in breast cancer lymphangiogenesis. For instance, nectin-4 promoted lymphangiogenesis through the CXCR4/CXCL12-LYVE-1 axis [45]. Lysyl oxidase-like protein 2 enhanced LECs invasion and lymphatic vessel formation via the activation of AKT-Snail and ERK pathways directly, or stimulated VEGF-C and CXCL12 secretion by tumor-associated fibroblasts [46]. However, their exact role needs to be further explored, as studies are increasingly demonstrating that tumor lymphangiogenesis shows high heterogeneity in metabolic mechanism, functional plasticity, and cellular origins [32,47].

The majority of tumor LECs sprout from pre-existing lymphatic vessels, while a few LECs have been found to originate from bone marrow-derived cell progenitors [35,36] (Figure 2b). For example, markers of myeloid lymphatic endothelial cell progenitors (M-LECPs) were found to be expressed on a portion of lymphatic vessels in breast cancer tissue, but not in normal breast tissue, and M-LECPs co-expressed high levels of PROX1, LYVE-1, podoplanin, and VEGFR-3 [36]. Current evidence for M-LECPs-derived LECs in tumor lymphangiogenesis could be observed in reference [36,48]. In addition, the insertion of TIE-2-expressing monocytes into lymphatic vessels was observed in breast cancer tissue but not in adjacent normal tissues [49]. Though the mechanisms underlying breast tumor lymphangiogenesis and LEC origin have not yet been fully elucidated, it is certain that tumor-associated lymphatic vessels may comprise endothelial cells with heterogeneous phenotypes according to different tumor microenvironments. Thus, LECs may share some markers in common with VECs or with some bone-marrow-derived cell progenitors.

## 3. Markers of LECs or Lymphatic Vessels

Ideally, during lymphangiogenesis assessment, positive markers should be specifically and sensitively expressed by LECs with a good signal-to-noise ratio compared to surrounding tissues, be easily detectable at the histological level, and must be resistant to biologic chemical agents during histological processing.

Due to its key role in lymphangiogenesis, PROX-1 is considered as a constitutive marker of LECs, located in the nuclei of all LECs in the physiologic and pathologic state [50] (Figure 2a). However, PROX-1 is also expressed by normal breast ductal epithelial cells and tumor cells. Studies showed that the expression of PROX-1 in breast tumor cells could reduce the MMP14-dependent invasiveness of the tumor cells [51], and *PROX-1* may be a suppressor gene in breast tumor cells [52]. Thus, PROX-1 alone is unsuitable as a specific marker for LVD and LVI assessments in breast cancer, but it is really an indispensable marker in identifying mechanisms underlying tumor lymphangiogenesis and origins of LEC when combined with other markers [36,49].

VEGFR-3, the receptor for VEGF-C and VEGF-D, is usually co-expressed on the surfaces of endothelial precursor cells and LECs with PROX-1, podoplanin, and LYVE-1 [53,54] (Figure 2a). VEGFR-3 is a membrane-anchored tyrosine kinase that is initially expressed on VECs during embryogenesis and early postnatal development but is restricted to LECs and some high endothelial venules during adulthood [54]. The key role of the VEGF-C/VEGF-D/VEGFR-3 signaling axis in lymphangiogenesis is currently unquestionable. However, the expression of VEGFR-3 is up-regulated on VECs of actively angiogenic blood vessels in breast cancer [55], which in turn leads to its poor lymphatic specificity. In addition, expression of VEGFR-3 is higher in tumor cells than in normal glandular cells [56,57].

LYVE-1 is a homologue of the CD44 hyaluronan receptor and an integral membrane glycoprotein involved in cell interactions [58]. LYVE-1 may not be essential for normal lymphatic development, as *LYVE-1*-gene-targeted mice develop normally and exhibit a functional network of lymphatic vessels [59]. The expression of LYVE-1 in normal breast ductal epithelial cells and tumor cells is not observed. Instead, LYVE-1 is selectively expressed on the overlapping flaps of LECs in initial lymphatic capillaries (Figure 2a) and significantly contributes to the transmigration of cells into lymphatics [60,61]. After its activation during cells transit, LYVE-1 can be endocytosed into or shed from LECs [62]. Thus, LYVE-1 on lymphatic capillaries presents the instability in immunostainings, and is absent on new single LECs or LECs bundles, which makes it inappropriate for the identification of intratumoral lymphangiogenesis. For example, Van der Auwera et al. [63] demonstrated that LYVE-1 showed weak or no immunoreactivity on intratumoral LECs.

**Figure 3:**
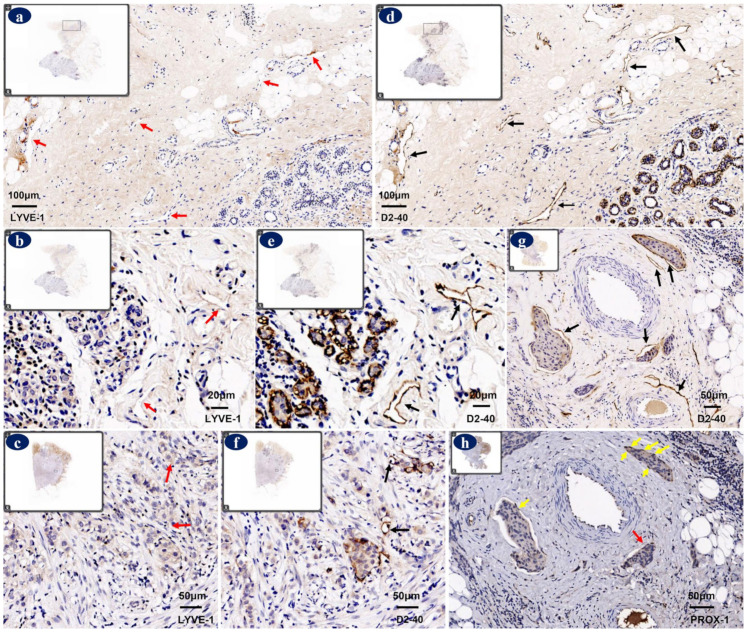
Immunoreactivity of lymphatic vessel endothelial hyaluronan receptor-1 (LYVE-1), D2-40, and prospero homeobox gene protein 1 (PROX-1) in breast cancer tissues. D2-40 showed higher sensitivity in identifying lymphatic endothelial cells (LECs) than anti-LYVE-1 antibody. The anti-LYVE-1 antibody showed weak or no immunoreactivity on lymphatics (red arrows) that were positive for D2-40 (black arrows) in periphery normal tissue ((**a**,**d**), 100×), peritumoral stroma ((**b**,**e**), 400×), and intratumoral tissue ((**c**,**f**), 200×). D2-40-positive stainings were localized on membrane of LECs (black arrows) ((**g**), 200×). PROX-1-positive stainings were localized in nuclei of LECs (yellow arrows) ((**h**), 200×). The lymphatic vessel seemed to be negative in PROX-1 staining (red arrow), because the nuclei of the lymphatic vessel were not present at this section ((**h**), 200×). Protocols for immunohistochemistry stainings were stated in Appendix A and the antibodies used were shown in Table A1.

Podoplanin is a transmembrane glycoprotein first detected on podocytes, which is also expressed on the luminal surface of LECs (Figure 2a), but not on VECs [64]. Podoplanin-null mice have defects in lymphatic vessels formation, which results in diminished lymphatic transport, congenital lymphedema, and the dilation of lymphatic vessels [65]; podoplanin-positive lymphatics were showed to be rate-limiting for breast cancer metastasis [66]. Stacker et al. [67] showed that podoplanin seemed to be expressed on small lymphatic vessels but not on lymphatic vessels covered with SMCs. D2-40 is one of the most widely used commercial monoclonal antibodies that binds to a fixation-resistant epitope of podoplanin [68]. It was reported that D2-40 showed higher sensitivity in distinguishing lymphatics than PROX-1 and LYVE-1 in breast cancer tissue [63,69,70] (Figure 3). D2-40 showed the strongest immunoreactivity in both intratumoral and peritumoral LECs, of D2-40 positive intratumoral vessels, 35.1% were positive for PROX-1 and 37.9% showed weak positive for LYVE-1 [63]. Notably, D2-40 is also positive in macrophages, cancer-associated fibroblasts [42,71], and myoepithelial cells [72] in breast tissue. Therefore, myoepithelial cell makers should be combined with D2-40 to differentiate LVI from ductal carcinoma in situ (DCIS).

Other molecules with poor specificity have limited role in identifying lymphatics. For instance, CD31 is a pan-endothelial marker expressed on the LECs and VECs, but it is found to be expressed more strongly in LYVE-1-negative VECs than in LYVE-1-positive LECs [73]. Neuropilin (NRP) 2 is not only a receptor for semaphorins in nerve tissue, but also a co-receptor of VEGFR-3 on LECs, which plays an important role in lymphangiogenesis via VEGF-C/NRP-2/VEGFR-3 axis [74]. However, NRP-2 is also expressed on breast cancer cells [75] and tumor-initiating cells [76]. In summary, all these markers used in combination reasonably with other biomarkers contribute to research in tumor lymphangiogenesis (Table 1). D2-40, LYVE-1, and PROX-1 are recognized LEC markers in human breast cancer, among which D2-40 has the best performance in lymphangiogenesis assessment from the current study. Meanwhile, with the intensive study of lymphatic structure and function, novel lymphatic markers are expected to be discovered in the near future.

## 4. Morphology of Tumor-Associated Lymphatic Vessels

**Figure 4:**
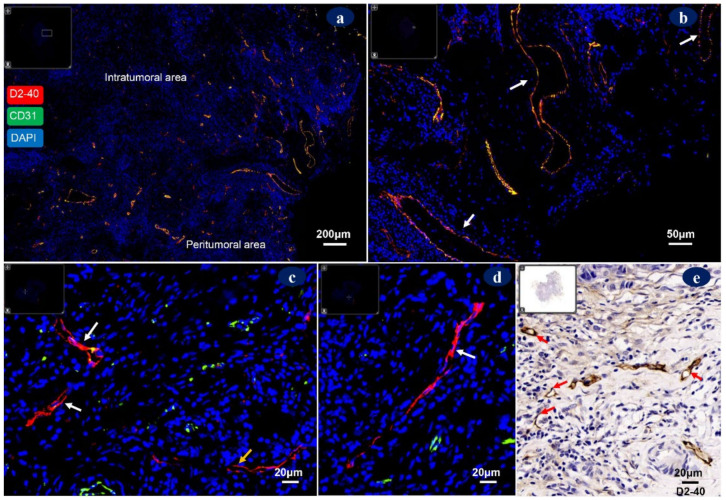
Double D2-40/CD31 immunofluorescence labeling was utilized to visualize distribution pattern and morphology of tumor-associated lymphatic vessels in breast cancer tissues. (**a**) Continuous D2-40-positive and discontinuous CD31-positive lymph vessels are mainly present in peritumoral area, but are negligible in intratumoral area (50×). (**b**) D2-40-positive large dilated lymphatic vessels in peritumoral area (white arrows, 200×). (**c**) D2-40-positive collapsed vessels (white arrows) and long narrow lymphatic vessel (yellow arrow, 400×) in intratumoral areas. (**d**) D2-40-positive lymphatic endothelial bundle (white arrow, 400×) in intratumoral areas. (**e**) D2-40-positive tumor-associated lymphatic endothelial cells (red arrows, 400×) in intratumoral areas. Protocols for immunofluorescence and immunohistochemistry stainings were stated in Appendix A and the antibodies used were shown in Table A1.

Tumor-associated lymphatic vessels exhibit highly heterogeneous morphology and function when compared to normal vessels. Dysregulated lymphatic vessels are organized in a non-hierarchical manner and consist of leaky vessels with irregular distributions. The role of intratumoral vs. peritumoral lymphatic vessels in the development of breast cancer has long been controversial. In peritumoral stroma, lymphatic vessels appear to be inflamed and dilated, which serve as the functional route for cancer-cell dissemination (Figure 4a,b). By contrast, the existence of lymphatic vessels in intratumoral areas is ambiguous, and the majority of the lymphatic vessels in intratumoral areas are sparse, dysfunctional, collapsed vessels with a long narrow or atretic shape (Figure 4c), which is unfavorable for lymph draining or cell migration [90]. These destructing lymphatics may contribute to increasing the compressive stress within tumor areas [91,92]. Some studies have even demonstrated that lymphangiogenesis is not evident in breast tissues and that breast cancer cells might invade and destroy intratumoral lymph vessels rather than stimulating them, and metastasis is mainly via pre-existing lymphatics [92,93].

However, LECs are actively involved in tumor cell growth and invasion, antitumor immunity, and tumor microenvironment remodeling [13,14,15]. We conjecture that the cancer-promoting role of intratumoral LECs could have been underestimated due to differences in assessments. Thus, more research is required to clarify the function of intratumoral lymphangiogenesis. A tumor-associated lymphatic vessels unit should preferably consider single immunoreactive LEC and positive LECs clusters or bundles separate from other lymphatic vessels (Figure 4d) irrespective of lumen status, but positive LECs of large vessels with BM were not included. It was demonstrated that in primary breast tumors, lymphatic vessels mainly present at the peritumoral stroma and only occasionally present in tumor nests and tumor-associated stroma, and LVD within tumor nests had no relationship with metastasis [19]. Whereas, Niemiec et al. [18] demonstrated that a high intratumoral LVD and LVI were adverse factors for disease-free survival in pN0/chemotherapy/trastuzumab-naïve patients.

**Figure 5:**
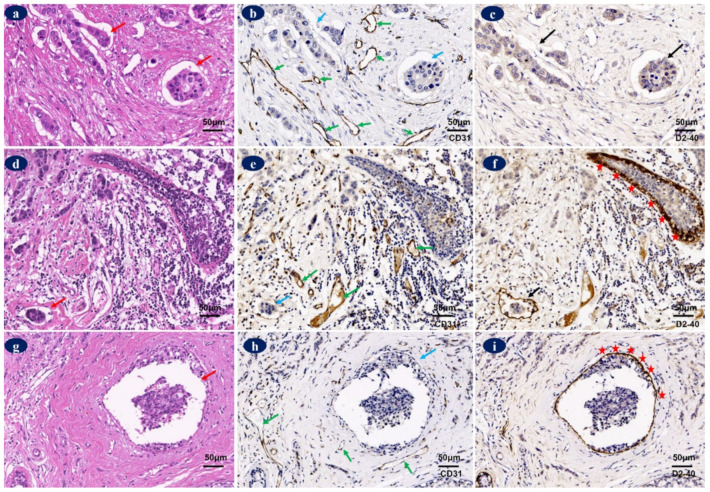
D2-40/CD31 immunohistochemistry staining was utilized to distinguish between lymphatic vessel invasion (LVI) and clefts, blood vessels invasion, or ductal carcinoma in situ in breast cancer tissues. (**a**) Clefts in HE (red arrows). (**b**) CD31-negative clefts (blue arrows) and CD31-positive blood vessels (green arrows). (**c**) D2-40-negative clefts (black arrows). (**d**) LVI in HE staining (red arrow). (**e**) Discontinuous CD31-positive LVI (blue arrow) and continuous CD31-positive blood vessels (green arrows). (**f**) Continuous D2-40-positive LVI (black arrow) and D2-40-positive myoepithelial cells (red stars). (**g**) Ductal carcinoma in situ (DCIS) in HE staining (red arrow). (**h**) CD31-negative DCIS (blue arrow) and CD31-positive blood vessels (green arrows). (**i**) D2-40-positive myoepithelial layer of the DCIS (red stars). (200×). Protocols for HE and immunohistochemistry stainings were stated in Appendix A and the antibodies used were shown in Table A1.

Another pathologic phenomenon, peritumoral cleft, was defined as clear spaces without a ECs layer that separate tumor cells from the stroma (Figure 5a–c). It has long been considered as fibroblasts retraction [94] or another pathogenesis, such as the situation when ECs shed from the vessel wall during improper tissue processing. Kos et al. [94] found that peritumoral clefts occurred in 92% of invasive ductal breast carcinomas and were not associated with lymphangiogenesis. However, some researchers have suggested that peritumoral clefts seemed to be immature unfinished lymphatic channels with prognostic value or a reflection of epithelial–stromal interaction during lymphangiogenesis [95,96].

## 5. Lymphatic Vessel Density (LVD)

The first international consensus on the methodology of lymphangiogenesis quantification suggested that the extent of tumor lymphangiogenesis could be quantified according to the LVD or lymphatic endothelial proliferation [97]. The LVD is a commonly used parameter in research (Table 2), based on the assumption that a high LVD might indicate an increased risk of cancer cell intravasation and intensive communication between cancer cells and LECs, which plays an important role in tumor progression [98]. However, some studies have yielded inconsistent conclusions regarding the prognostic value of the LVD. On the one hand, Zhang et al. [16] showed that a high LVD and LVI were both unfavorable prognostic factors in breast cancer, and the peritumoral LVD was higher than the intratumoral LVD (77.9% vs. 40.07%), while both the peritumoral and intratumoral LVD were positively correlated with lymph node metastases. Chen et al. [66] showed that a reduced LVD could up-regulate the frequency of intratumoral macrophages and reduce lung metastasis in breast cancer. On the other hand, Britto et al. [99] indicated that though D2-40 improves the diagnostic accuracy of the LVD and LVI, neither the LVD nor LVI could be used as a predictor of sentinel lymph-node metastasis. Furthermore, Niemiec et al. [18] indicated that a high LVD might identify a low progression risk in pN+/chemotherapy-treated patients. That is to say, chemotherapy-treated breast cancer patients with a high LVD showed better survival. This is theoretically explicable; the tumor antigens, immune cells, and drug transported via lymphatic vessels and blood vessels may play a beneficial role in cancer therapy [17,100]. For example, Kruger et al. [100] showed that a high baseline microvessel density was significantly associated with the response to bevacizumab. However, tumor vasculatures are dysfunctional, leading to immunosuppression and therapy resistance in the vast majority of cases, and normalizing the tumor vasculature can optimize drug uptake [90].

A range of confounding factors may be responsible for the equivocal conclusions (Table 2). Firstly, different endothelial markers were utilized across the studies. Secondly, there is currently no clinical consensus for LVD assessment. The LVD is usually assessed using the Weidner method [101], which was first proposed in 1991 for quantifying blood microvessel density. Hotspot areas with the most lymphatic vessels are firstly chosen at low magnification; then vessels are counted at high magnification in representative fields within the hotspots [101,102]. However, the representative fields used in research were chosen with different magnifications, number, and acreage. Moreover, vessel counts were recorded with the maximum, median, or total counts resulting in varied cut-off values.

**Table 2:** Studies on the relationship between the lymphatic vessel density (LVD) and breast cancer survival over the last decade.

First Author	(No.) Patients	Antibody (Corp, Dilution)	LVD Evaluation Areas	Counts (Magnification)	Cutoff Value	High LVD
Norhisham et al. [83]	(58) Breast carcinoma	D2-40 (Dako, 1:100) CD 34 (Dako, 1:100)	Intra- and peritumoral	Sum of vessels/Sum of area (100×)	Median value	Distant metastasis (peri-LVD)
Niemiec et al. [18]	(139) pT1-2N0M0 IDC chemotherapy-naive	D2-40 (Cell Marque, 1:100) CD34 (QBEnd 10, 1:50)	Intra- and peritumoral, peripheral	The highest of 20 hotspots (100×)	Minimum *p*-value	Poor DFS and MFS
Niemiec et al. [18]	(215) pT1-3N+M0 IDC chemotherapy-treated	D2-40 (Cell Marque, 1:100) CD34 (QBEnd 10, 1:50)	Intra- and peritumoral, peripheral	The highest of 20 hotspots (100×)	Minimum *p*-value	Favorable DFS and MFS
Abe et al. [87]	(91) IDC	D2-40 (1:100)	NA	Mean value of 5 hotspots (200×)	Mean value	Poor OS and DFS
Wahal et al. [84]	(30) Invasive breast carcinoma	D2-40 (Dako, NA) CD31 (Dako, NA)	Intratumoral, peritumoral	Mean value of 5 hotspots (NA)	NA	High lymph node ratio
Zhao et al. [103]	(73) IDC	D2-40 (Signet, 1:25)	Intratumoral, peritumoral	Mean value of 5 hotspots (NA)	Median value	Poor OS and DFS (peri-LVD)
Mohammed et al. [86]	(197) N0 Basal-like BC (200) N0 Non-basal-like BC	D2-40 (AngioBio, 1:100) CD34 (Serotec, 1:500) CD31 (Dako, 1:100)	Whole section	Sum of vessels/sum of area (100×)	NA	No association with 20-year OS

Abbreviations: Corp: corporation; DFS, disease-free survival; IDC, invasive ductal breast cancer; MFS, metastasis-free survival; NA, not available; OS, overall survival; peri-LVD, peritumoral lymphatic vessel density.

## 6. Lymphatic Vessel Invasion (LVI)

Lymphovascular invasion is the presence of tumor emboli in lymphatic vessels or blood vessels implying an increased risk of dissemination in breast cancer [16,104,105]. Nevertheless, the independent prognostic value of LVI in breast cancer remains unclear, as in many cases, lymphovascular invasion has been assessed through hematoxylin–eosin (HE) stains, which cannot distinguish between LVI and clefts, blood vessels invasion, or DCIS [70,83,106,107] (Figure 5). The concordance of lymphovascular invasion assessment between pathologists based on HE staining is variable, ranging from a median of 0.86 (0.54, 0.99) in a lymphovascular-invasion-positive group to 0.93 (0.52, 1.0) in the lymphovascular-invasion-negative group [106]. An immunohistochemistry (IHC) staining with endothelial markers can accurately distinguish lymphatic vessels [70,105,107]. Abbasi et al. [108] demonstrated that the kappa coefficient between HE and D2-40 staining for detecting lymphovascular invasion was 0.078; and the specificity and negative predictive values of HE staining were 66% and 54.8%, respectively.

Even so, there exists contradictory findings; some studies have demonstrated that the LVI was associated with poor outcomes [70,83] (Table 3). It was reported that though the blood vessel density was higher than the LVD in breast cancer, tumor emboli predominantly occurred in lymphatic vessels [83,85]. However, Kos et al. [94] demonstrated that in patients with axillary lymph node metastasis, the peritumoral LVI was twice and the intratumoral LVI was five times more than that in patients without lymph node metastasis. Fujii et al. [109] suggested that it was blood vessel invasion, not the LVI, which indicated high biological aggressiveness. One cause for the contradiction is the variation in the markers and stain methods utilized in detecting lymphovascular invasion. Another cause is the inherent microfocal nature of lymphovascular invasion. It was observed that only 20% lymphovascular invasions are consistently present in the tumor tissue; others present focal or inconsistent foci that are highly likely to be missed on tissue sections, leading to false negatives [106]. It is perhaps for this reason that IHC is not routinely used for the initial screening for lymphovascular invasion in most clinical institutions. Thus, the above factors limit the clinical utility of the LVI as a robust predictor for breast cancer.

## 7. Lymphangiogenesis in the Era of Precision Medicine

In the era of precision medicine, the treatment concept for early breast cancer has shifted from the maximum tolerated therapy to the minimum effective therapy [111]. Accurate risk stratification is essential for making a tailoring treatment to maximize survival while minimizing unnecessary interventions. The traditional prognostic tool, such as TNM staging, shows little or no prognostic value in early breast cancer without lymph node involvement or distant metastases. In this case, the characteristics of the tumor microenvironment, such as tumor lymphangiogenesis and angiogenesis, can be predictive or prognostic biomarkers, which well complements the TNM staging system and tumor molecular profiling. Compared with other molecular biotechnology, histomorphology has advantages in combining morphological characteristics with protein expression in tissue in situ and plays an irreplaceable role in lymphangiogenesis research.

In addition, the elucidation of the molecular mechanisms underlying the tumor lymphangiogenesis process will provide novel therapeutic strategies for anti-tumor immunomodulation. For instance, targeting VEGFR-3/-2 signaling pathways could be a potential strategy for combating lymphatic metastases [41,79]. Inhibiting NADPH oxidase 4 attenuated lymphangiogenesis in breast cancer [112], and the knockdown of lysyl oxidase-like protein 2 (LOXL2) suppressed lymphangiogenesis and lymph nodes metastases [46]. In addition, miR-128-3p may modulate the proliferation of LECs via directly targeting VEGF-C/VEGFR3 [113].

## 8. Conclusions

In summary, with the discovery of lymphatic markers, intensive studies on lymphangiogenesis reveal that LECs play an actively role in the invasion–metastasis cascade of breast cancer. However, we should bear in mind that tumor-associated lymphatic vessels comprise LECs with heterogeneous phenotypes, and there are no ideal markers that are restricted to the LECs or work robustly in various pathologic conditions hitherto. Thus, sensitive and specific LECs markers and standard lymphangiogenesis assessments are warranted to avoid controversial conclusions. In the meantime, in-depth research is progressively revealing the mechanisms and distinct roles of lymphangiogenesis in breast cancer progression, which is helping in the exploration of novel prognostic and predictive biomarkers and therapeutic targets for breast cancer.

## Figures and Tables

**Table 1:** The most commonly used lymphatic markers in breast cancer.

Markers	Property	Location on Lymphatics	Expressed on Other Cells	Relevant Research
PROX-1	Transcription factor	In nuclei of all LEC [50]	TEM [49], normal epithelial cell and tumor cell [51,52]	Lymphangiogenesis and cancer development [36,49,51,52]
VEGFR-3	Transmembrane protein	On cell membrane of LEC [54]	TEM [49], VEC [55,56], Tumor cell [56,57], MEC [56]	Lymphangiogenesis and cancer development [49,57,77,78], VEGFR-3 targeted therapy [41,79]
LYVE-1	Transmembrane protein	On overlapping flaps of LEC [21,23]	M-LECP [36], TEM [49], macrophage [80,81]	Lymphangiogenesis [36,49], cell migration [60,61], therapeutic responsiveness [80,81]
D2-40/podoplanin	Transmembrane glycoprotein	On cell membrane of LEC [64]	Macrophage [42], TEM [49], CAF [71], MEC [82]	Lymphangiogenesis and cancer development [42,49,66,71], LVD and LVI [18,63,83,84,85,86,87]
CD31	Transmembrane protein	On overlapping flaps of LEC and zipper-like junction [21,23]	VEC [23], megakaryocyte, Platelet, lymphocyte, etc. [88]	LVD and LVI [84,85,86]
Neuropilin-2 (NRP-2)	Transmembrane glycoprotein	Cell membrane of LEC [74]	Tumor cell [75], tumor-initiating cell [76]	Lymphangiogenesis/cancer development [74,75,76], NRP-2 targeted therapy [89]

Abbreviations: CAF, cancer-associated fibroblasts; LEC, lymphatic endothelial cells; LVD, lymphatic vessel density; LVI, lymphatic vessel invasion; LYVE-1, lymphatic vessel endothelial hyaluronan receptor-1; MEC, myoepithelial cell; M-LECP, myeloid lymphatic endothelial cell progenitor; PROX-1, prospero homeobox gene protein 1; TEM, TIE-2 expressing monocyte; VEC, vascular endothelial cells; VEGFR-3, vascular endothelial growth factor receptor-3.

**Table 3:** Studies on the relationship between the lymphatic vessel invasion (LVI) and breast cancer survival over the last decade.

First Author	(No.) Patients	Antibody (Corp, Dilution)	LVI Rate	High LVI and Survival
Kos et al. [94]	(100) Invasive breast carcinoma	D2-40 (Dako, 1:100), CD 34 (Dako, 1:50), Vimentin (Dako, 1:50)	13% * 43% ^#^	Higher axillary metastases
Niemiec et al. [18]	(139) pT1-2N0M0 IDC chemotherapy-naive	D2-40 (Cell Marque, 1:100), CD34 (QBEnd 10, 1:50)	5.8%	Poor DFS
Niemiec et al. [18]	(215) pT1-2N0M0 IDC chemotherapy-naive	D2-40 (Cell Marque, 1:100), CD34 (QBEnd 10, 1:50)	22.8%	No association with DFS and MFS
He et al. [110]	(255) IDC	D2-40 (Covance, 1:100)	25.1%	Poor DFS
Fadia et al. [70]	(360) IDC	D2-40 (Covance, 1:100), Factor VIII (Leica, 1:100)	35.3%	Poor cancer specific survival
Fujii et al. [109]	(263) Primary breast cancer	NA	14.3%	No association with RFS and OS
Zhao et al. [103]	(73) IDC	D2-40 (Signet, 1:25)	34.2%	No association with DFS and OS
Mohammed et al. [85]	(1005) N0 Invasive breast carcinoma	D2-40 (AngioBio, 1:100), CD34 (Serotec, 1:500), CD31 (Dako, 1:100)	21.2%	Poor 20-year DFI and 20-year OS
Mohammed et al. [86]	(197) N0 Basal-like BC, (200) N0 Non-basal-like BC	D2-40 (AngioBio, 1:100), CD34 (Serotec, 1:500), CD31 (Dako, 1:100)	22.9%	Poor 20-year OS

Abbreviations: BC, breast cancer; DFI, disease-free interval; DFS, disease-free survival; IDC, invasive ductal carcinoma; MFS, metastasis-free survival; OS, overall survival; RFS, relapse-free survival. * intratumoral LVI rate, ^#^ peritumoral LVI rate.

## Data Availability

Not applicable.

## Appendix A. Protocols

Parallel hematoxilin-eosin (HE), immunohistochemistry (IHC), and immunofluorescence (IF) stainings were performed in consecutive sections derived from patients with invasive breast cancer.

IHC staining: Firstly, serial paraffin sections of 4 μm thickness were deparaffinized in xylene (15 min each) and rehydrated in a series of descending ethanol concentrations (5 min each). Then antigen retrieval was performed in sodium citrate buffer (Ph 6.0) in a microwave (10 min at full power followed by 10 min at low power). And endogenous peroxidase activity was blocked with 3% H_2_O_2_ (room temperature for 25 min). Secondly, sections were incubated with the primary antibodies overnight at 4℃. Unbound primary antibody was washed with PBS (pH 7.4, 3 times, 5 min each) prior to the addition of HRP-labeled polymer (room temperature for 50 min). Finally, visualized with 3, 3′-diaminobenzidine chromogens (DAB), counterstained with hematoxylin (3 min), dehydrated in a series of ascending ethanol concentrations and xylene.

IF staining: Firstly, serial paraffin sections of 4 μm thickness were deparaffinized in xylene (15 min each) and rehydrated in a series of descending ethanol concentrations (5 min each). Then antigen retrieval was performed in sodium citrate buffer (Ph 6.0) in a microwave (10 min at full power followed by 10 min at low power). Secondly, sections were incubated with the anti-CD 31 antibody overnight at 4 °C. Unbound primary antibody was washed with PBS (pH 7.4, 3 times, 5 min each) prior to the addition of HRP-labeled polymer (room temperature for 50 min), followed by PBS (pH 7.4, 3 times, 5 min each) and incubated with TSA-488 (room temperature for 30 min). Then antigen retrieval was repeated as mentioned above. Thirdly, sections were incubated with the D2-40 overnight at 4 °C and incubated with CY3-tyramide (room temperature for 50 min). Finally, counterstained with DAPI (room temperature for 10 min).

**Table A1:** Antibodies used for immunohistochemistry and immunofluorescent stains.

Antibody	Mainly Expressed	Type	Dilution	Corp
Primary Antibodies				
LYVE-1	Lymph endothelia	Rabbit, monoclonal	1:200	Bioss
CD31	Endothelia	Rabbit, monoclonal	1:2000	Abcam
D2-40	Lymph endothelia	Rabbit, monoclonal	1:200	Abways
PROX-1	Lymph endothelia	Rabbit, monoclonal	1:500	Abcam
Secondary Antibodies				
Goat anti-rabbit-HRP antibody			Ready-to-use	DAKO
TSA-488			1:4000	Proteintech Group
CY3-Goat anti-rabbit tyramide			1:500	Jackson
DAB			1:100	DAKO
DAPI			1:100	Solarbio
